# Association between the skeletal muscle-to-visceral fat ratio and kidney stones: a cross-sectional study

**DOI:** 10.3389/fnut.2025.1549047

**Published:** 2025-05-08

**Authors:** Yuan-Zhuo Du, Jia-Qing Yang, Jian Tang, Chi-Teng Zhang, Yi-Fu Liu

**Affiliations:** ^1^Department of Urology, The First Affiliated Hospital, Jiangxi Medical College, Nanchang University, Nanchang, China; ^2^The Second Affiliated Hospital, Department of Urology, Hengyang Medical School, University of South China, Hengyang, Hunan, China

**Keywords:** skeletal muscle-to-visceral fat ratio, kidney stones, NHANES, cross-sectional study, skeletal muscle, visceral fat

## Abstract

**Objective:**

Prior research has suggested links between skeletal muscle mass and visceral fat volume with kidney stone formation. However, the link between the skeletal muscle-to-visceral fat ratio (SVR) and kidney stone risk remains to be clarified. This study aims to explore the relationship between SVR and the risk of kidney stones, analyzing data from the National Health and Nutrition Examination Survey (NHANES).

**Methods:**

The research encompassed 8,522 individuals from NHANES surveys from 2011 to 2018. Kidney stones were diagnosed through a standardized questionnaire, and SVR was calculated using dual-energy X-ray absorptiometry (DXA). Participants were grouped into quartiles based on their SVR. All data underwent weighting according to official guidelines. Logistic regression models assessed the correlation between SVR and kidney stone incidence, and subgroup analysis was employed to investigate its stability.

**Results:**

Among the participants, 675 individuals, representing 8.73%, received a diagnosis of kidney stones, with an average age of 39.29 years (±0.28). Findings indicate that lower SVR correlates with increased kidney stone risk. Within the comprehensively adjusted multivariate model, compared to the lowest SVR quartile, the second, third, and fourth quartiles demonstrated significantly reduced risks, with ORs of 0.63 (95% CI = 0.47–0.84), 0.57 (95% CI = 0.42–0.79), and 0.39 (95% CI = 0.25–0.61), respectively. Restricted cubic spline (RCS) regression models demonstrated a non-linear relationship between SVR and kidney stone risk. The subgroup analysis demonstrated no significant differences in weighted associations across subgroups (interaction *p*-value > 0.05), except for BMI, which had a significant interaction (interaction *p*-value < 0.05).

**Conclusion:**

The findings underscore that lower SVR correlates with increased kidney stone risk, a relationship that remains consistent across most demographics.

## 1 Introduction

Kidney stone disease, a prevalent urological disorder, has an escalating global incidence, representing a significant public health issue (1, 2). The prevalence of kidney stones varies markedly across regions, with rates as high as 12–18% (3, 4). Kidney stones not only inflict severe pain but also lead to renal impairment and other complications (2, 5), underscoring the importance of researching preventive and early intervention strategies.

In recent years, research has expanded beyond traditional risk factors like diet, genetics, and environmental influences to include the influence of body composition, particularly the roles of body fat distribution and skeletal muscle mass in the formation of kidney stones (6, 7). Visceral fat buildup is recognized as a risk factor for various metabolic diseases, including cardiovascular diseases (CVD) and type 2 diabetes, and may indirectly participate in kidney stone formation by altering metabolic and endocrine environments (7–9). On the other hand, a higher skeletal muscle mass is generally considered a protective factor for health, enhancing metabolic well-being and reducing the risk of various illnesses (10–12).

While previous research has examined the individual associations between skeletal muscle mass and visceral fat area with kidney stone risk, the collective impact of the skeletal muscle-to-visceral fat ratio (SVR) remains underexplored. SVR, as an integrated body composition marker, may provide a more precise reflection of an individual’s health status and disease risk (13). This study utilizes large-scale data from the National Health and Nutrition Examination Survey (NHANES), applying dual-energy X-ray absorptiometry (DXA) to precisely determine SVR and investigate its correlation with kidney stone risk. We hypothesize that an elevated SVR correlates with a reduced likelihood of developing kidney stones, and this association remains consistent across different groups.

## 2 Methods

### 2.1 Study population

**Figure 1:**
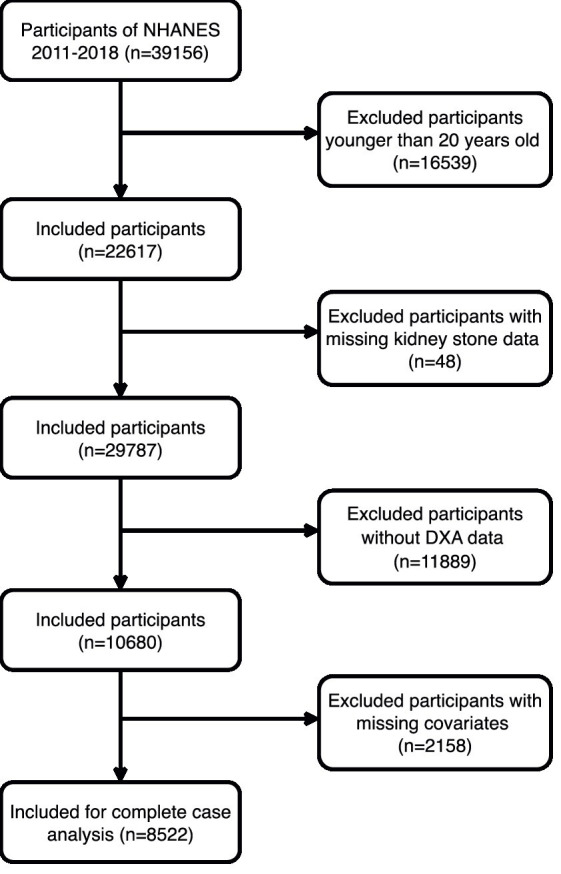
The participant flow diagram.

This cross-sectional study analyzed NHANES database data from 2011 to 2018, updated every 2 years. Conducted post-approval from the National Center for Health Statistics’ Ethics Review Board and with participant consent, the study filtered 8,522 suitable subjects from an initial 39,156 based on inclusion and exclusion criteria. Exclusions were individuals under 20 years (*n* = 16,539), missing kidney stone questionnaire data (*n* = 48), those not undergoing DXA (*n* = 11,889), and lacking essential covariate information (*n* = 2,158). Refer to Figure 1 for the detailed selection process.

### 2.2 DXA measurements and definition of SVR

Whole-body DXA scans were conducted on non-pregnant participants aged 8–59, excluding recent users of radiographic contrast agents such as barium or those over 450 pounds or 6 feet 5 inches tall. All DXA scans were strictly quality-controlled to ensure accuracy. Skeletal muscle mass was estimated from the total lean mass of the limbs (arms and legs), and the SVR was calculated as the ratio of non-abdominal lean mass to visceral fat area (kg/cm^2^).

Visceral fat area was specifically measured using the Hologic APEX software during DXA scans. The visceral adipose tissue (VAT) mass and volume were determined at the approximate intervertebral space of L4 and L5 vertebrae, which is located within the abdominal cavity. The boundaries of the visceral fat area were automatically defined by the Hologic software based on this region. Participants were then categorized into quartiles based on their SVR for analysis.

### 2.3 Diagnosis of kidney stones

Kidney stone diagnoses relied on participants answering “Have you ever had kidney stones?” The reliability of self-reported kidney stone history is supported by prior research (14).

### 2.4 Definition of covariates

This research includes a variety of covariates associated with SVR and kidney stone risk, divided into three main categories: demographic indicators, lifestyle factors, and health status. Demographic indicators comprise age, sex, race, marital status, level of education, and poverty income ratios (PIR). Lifestyle factors cover alcohol consumption (categorized into lifelong abstainers who drank less than 12 times, former drinkers who drank 12 times or more but abstained in the past year, and current drinkers who have consumed alcohol 12 times or more in their lifetime and at least once in the last year) (15), smoking status (based on whether an individual has smoked over 100 cigarettes in their lifetime), sedentary behavior (daily sitting time exceeding 5 h), and physical activity level (assessed by the duration of moderate to intense activity lasting at least 10 min per week beyond regular work and commuting, with less than 10 min classified as inactive) (16). Health indicators are collected using standardized questionnaires and clinical evaluations, including body mass index (BMI), estimated Glomerular Filtration Rate (eGFR), diabetes, hypertension, hyperlipidemia, and the prevalence of CVD.

### 2.5 Statistical analysis

Statistical analyses incorporated NHANES’s sampling weights. Continuous variables are reported as weighted means and standard errors, while categorical variables are shown as weighted counts and proportions. Analyses involved weighted linear regression and chi-square tests. Multivariable logistic regression models were used to investigate the association between SVR and the incidence of kidney stones, providing odds ratios (ORs) and 95% confidence intervals (CIs). Models were stratified by covariates: unadjusted crude model; Model 1 adjusted for basic demographics; Model 2 further considered lifestyle and health status. Restricted cubic spline (RCS) regression models examined the dose–response relationship between SVR and kidney stone risk, with subgroup analyses for robustness. All analyses were performed using R software (version 4.3.2), with a significance level set at *p* < 0.05.

## 3 Results

### 3.1 Baseline characteristics of participants

From the 2011–2018 NHANES database across four cycles, a total of 8,522 participants were enrolled and grouped into quartiles based on their SVR. Table 1 indicates that the average age of the participants was 39.06 ± 0.28 years. Among them, 675 were diagnosed with kidney stones. Participants in the higher SVR quartiles typically were younger, predominantly male and non-Hispanic Black, more often unmarried with higher educational attainment and PIR, lower BMIs, and were more likely to be non-smokers and current drinkers. They also tended to lead more active lifestyles, sit less, have higher eGFR, and have lower rates of hypertension, diabetes, hyperlipidemia, and CVD. In contrast, those in the lower SVR quartiles had a higher incidence of kidney stones.

**Table 1:** Baseline characteristics of the study population by quartiles of SVR.

Variable	SVR (kg/m^2^) quartiles					*P*-value
	Overall	Q1 (≤0.17)	Q2 (0.18–0.24)	Q3 (0.25–0.38)	Q4 (≥0.39)	
Age, y, mean (SE)	39.29 (0.28)	46.62 (0.27)	41.76 (0.29)	37.39 (0.42)	31.61 (0.35)	<0.0001
Sex, *n* (%)						<0.0001
Female	4,242 (48.96)	1,502 (67.47)	1,001 (44.18)	909 (43.13)	830 (41.46)	
Male	4,280 (51.04)	621 (32.53)	1,140 (55.82)	1,220 (56.87)	1,299 (58.54)	
Race, *n* (%)						<0.0001
Mexican American	1,233 (9.93)	501 (13.88)	369 (12.26)	239 (8.87)	124 (4.81)	
Non-Hispanic White	3,148 (63.50)	833 (66.21)	799 (63.25)	729 (61.15)	787 (63.40)	
Non-Hispanic Black	1742 (10.58)	182 (4.18)	346 (8.10)	512 (12.49)	702 (17.37)	
Other Hispanic	843 (6.85)	278 (7.82)	206 (6.53)	203 (7.13)	156 (5.93)	
Other Race	1,556 (9.15)	329 (7.90)	421 (9.86)	446 (10.36)	360 (8.48)	
Marital status, *n* (%)						<0.0001
Divorced/Separated/Widowed	1,190 (13.22)	462 (21.50)	304 (13.66)	246 (11.14)	178 (6.79)	
Married/Living with a partner	5,086 (62.26)	1,391 (66.23)	1,440 (69.50)	1,260 (62.12)	995 (51.41)	
Never married	2,246 (24.52)	270 (12.26)	397 (16.84)	623 (26.74)	956 (41.80)	
Education levels, *n* (%)						<0.0001
High school and below	3,236 (32.89)	969 (38.60)	862 (35.81)	740 (30.26)	665 (27.03)	
Above high school	5,286 (67.11)	1,154 (61.40)	1,279 (64.19)	1,389 (69.74)	1,464 (72.97)	
Poverty ratio, *n* (%)						0.03
<1.3	2,694 (22.47)	774 (24.91)	647 (21.39)	612 (20.04)	661 (23.54)	
1.3–3.5	3,092 (34.78)	742 (34.51)	800 (36.10)	768 (33.52)	782 (34.96)	
>3.5	2,736 (42.76)	607 (40.58)	694 (42.52)	749 (46.43)	686 (41.50)	
BMI, *n* (%)						<0.0001
<25	2,696 (31.43)	208 (7.77)	416 (17.87)	707 (32.59)	1,365 (66.61)	
25–29.99	2,732 (33.10)	652 (30.33)	806 (39.35)	776 (39.51)	498 (23.35)	
≥30	3,094 (35.47)	1,263 (61.89)	919 (42.79)	646 (27.90)	266 (10.04)	
Smoke, *n* (%)						<0.001
No	5,156 (59.04)	1,224 (53.55)	1,295 (59.32)	1,317 (60.38)	1,320 (62.76)	
Yes	3,366 (40.96)	899 (46.45)	846 (40.68)	812 (39.62)	809 (37.24)	
Alcohol user, *n* (%)						<0.0001
Never	1,082 (9.42)	379 (12.54)	244 (9.06)	239 (8.44)	220 (7.72)	
Former	769 (8.09)	269 (11.85)	213 (9.22)	164 (6.14)	123 (5.22)	
Now	6,671 (82.49)	1,475 (75.61)	1,684 (81.72)	1726 (85.42)	1786 (87.06)	
Moderate recreational activity, *n* (%)						<0.0001
No	3,712 (39.04)	1,202 (52.84)	1,025 (42.63)	841 (35.15)	644 (25.95)	
Yes	4,810 (60.96)	921 (47.16)	1,116 (57.37)	1,288 (64.85)	1,485 (74.05)	
Sitting time, *n* (%)						0.2
<5	3,086 (33.09)	778 (30.71)	797 (32.98)	758 (34.15)	753 (34.48)	
≥5	5,436 (66.91)	1,345 (69.29)	1,344 (67.02)	1,371 (65.85)	1,376 (65.52)	
Hypertension, *n* (%)						<0.0001
No	6,557 (77.96)	1,357 (64.07)	1,576 (73.72)	1722 (83.04)	1902 (90.62)	
Yes	1965 (22.04)	766 (35.93)	565 (26.28)	407 (16.96)	227 (9.38)	
Diabetes, *n* (%)						<0.0001
No	7,749 (92.83)	1738 (85.40)	1917 (91.09)	2016 (96.54)	2078 (98.10)	
Borderline	160 (1.71)	69 (2.89)	46 (2.21)	28 (0.96)	17 (0.79)	
Yes	613 (5.47)	316 (11.71)	178 (6.69)	85 (2.50)	34 (1.10)	
Hyperlipidemia, *n* (%)						<0.0001
No	3,209 (37.64)	351 (15.69)	591 (27.96)	892 (41.09)	1,375 (65.10)	
Yes	5,313 (62.36)	1772 (84.31)	1,550 (72.04)	1,237 (58.91)	754 (34.90)	
CVD, *n* (%)						<0.0001
No	8,438 (99.25)	2082 (98.29)	2,121 (99.24)	2,113 (99.62)	2,122 (99.82)	
Yes	84 (0.75)	41 (1.71)	20 (0.76)	16 (0.38)	7 (0.18)	
eGFR (mL/min), mean (SE)	101.54 (0.39)	98.03 (0.56)	100.23 (0.45)	102.37 (0.63)	105.44 (0.59)	<0.0001
Kidney stone, *n* (%)						<0.0001
No	7,847 (91.27)	1846 (84.95)	1960 (91.19)	2000 (92.99)	2041 (95.79)	
Yes	675 (8.73)	277 (15.05)	181 (8.81)	129 (7.01)	88 (4.21)	

SVR, skeletal muscle mass to visceral fat area ratio; eGFR, estimated glomerular filtration rate; BMI, body mass index; CVD, cardiovascular disease.

### 3.2 Association between SVR and kidney stones

**Figure 2:**
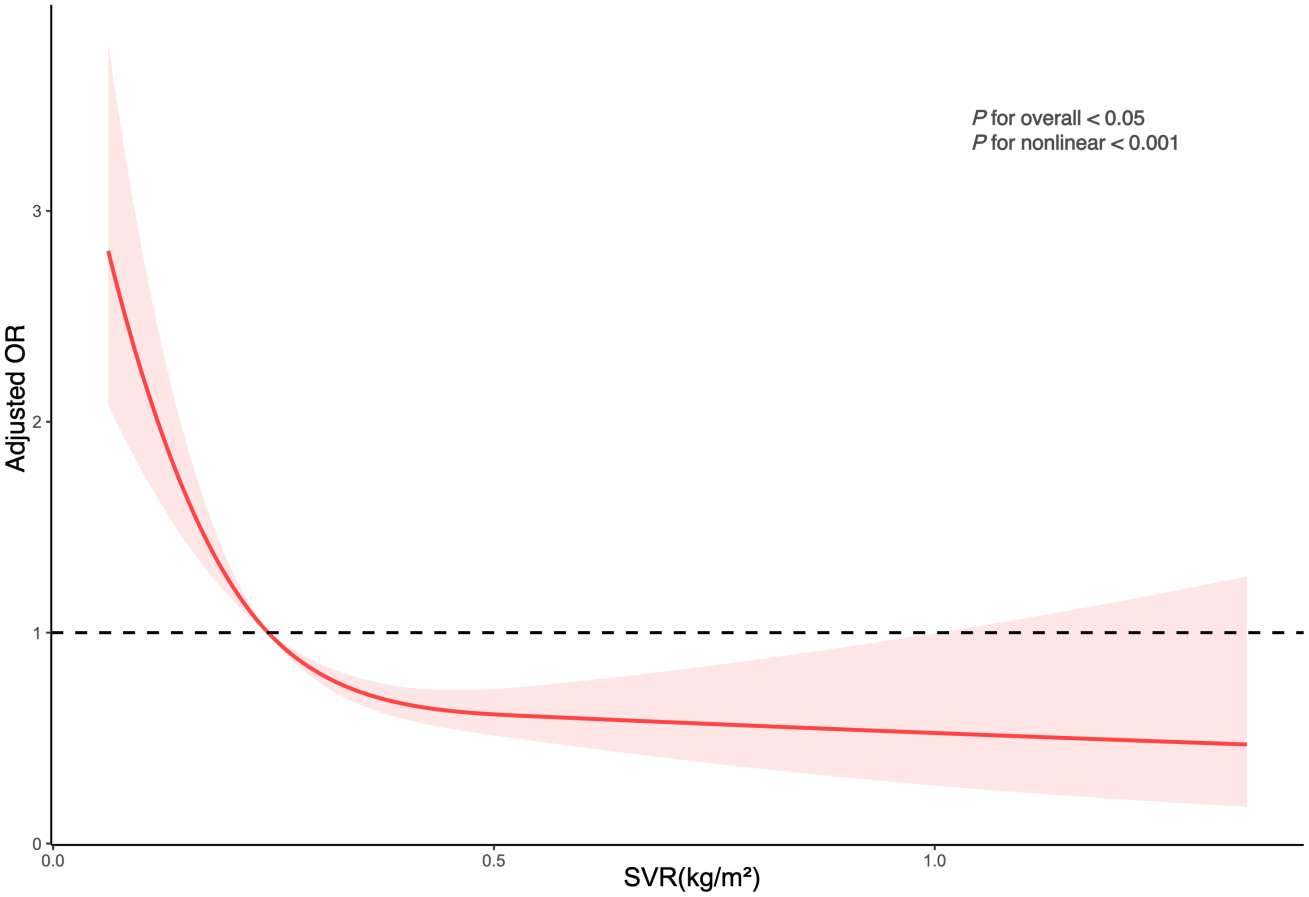
Depicts the association between SVR and kidney stone occurrences. The ORs, shown as solid lines, were adjusted for factors including age, sex, race, marital status, educational attainment, poverty index, BMI, smoking habits, alcohol consumption, recreational activities, sitting duration, eGFR, hypertension, diabetes, hyperlipidemia, and CVD. The corresponding 95% CIs are indicated by shaded regions.

The logistic regression analysis in Table 2 shows that for each unit increase in SVR, there was a significant decrease in kidney stone risk (OR = 0.03, 95% CI: 0.01–0.09, *p* < 0.0001). Even with adjustments for all covariates, this significant negative correlation persisted, with every incremental increase in SVR associated with a further decrease in risk (OR = 0.12, 95% CI: 0.03–0.52, *p* = 0.01). Relative to the bottom quartile of SVR, the second, third, and fourth quartiles had progressively lower risks of developing kidney stones (OR = 0.63, 95% CI: 0.47–0.84; OR = 0.57, 95% CI: 0.42–0.79; OR = 0.39, 95% CI: 0.25–0.61). These findings indicate that higher SVR is significantly associated with a reduced risk of kidney stones. The RCS regression analysis demonstrated a notable non-linear association between SVR and the risk of kidney stones, showing a pronounced decrease in risk with increasing SVR levels (Figure 2).

**Table 2:** Association of the quartiles of SVR with kidney stone.

Exposure	Crude model		Model 1		Model 2	
	OR (95% CI)	*p*-value	OR (95% CI)	*P*-value	OR (95% CI)	*P*-value
SVR	0.03 (0.01,0.09)	<0.0001	0.07 (0.02,0.23)	<0.0001	0.12 (0.03,0.52)	0.01
SVR quartile						
Q1 (≤0.17)	1 (Ref.)		1 (Ref.)		1 (Ref.)	
Q2 (0.18–0.24)	0.55 (0.42,0.71)	<0.0001	0.58 (0.44,0.76)	<0.001	0.63 (0.47,0.84)	0.003
Q3 (0.25–0.38)	0.43 (0.33,0.55)	<0.0001	0.50 (0.37,0.67)	<0.0001	0.57 (0.42,0.79)	0.001
Q4 (≥0.39)	0.25 (0.18,0.35)	<0.0001	0.32 (0.22,0.46)	<0.0001	0.39 (0.25,0.61)	<0.001
P for trend		<0.0001		<0.0001		<0.0001

Crude model: unadjusted model.

Model 1: adjusted for age, sex, race, education levels, marital status, poverty ratio.

Model 2: additionally adjusted for BMI, smoking, alcohol user, recreational activity, sitting time, eGFR, hypertension, diabetes, hyperlipidemia and CVD.

SVR, skeletal muscle mass to visceral fat area ratio; OR, odds ratio; CI, confidence interval.

### 3.3 Subgroup analysis

**Figure 3:**
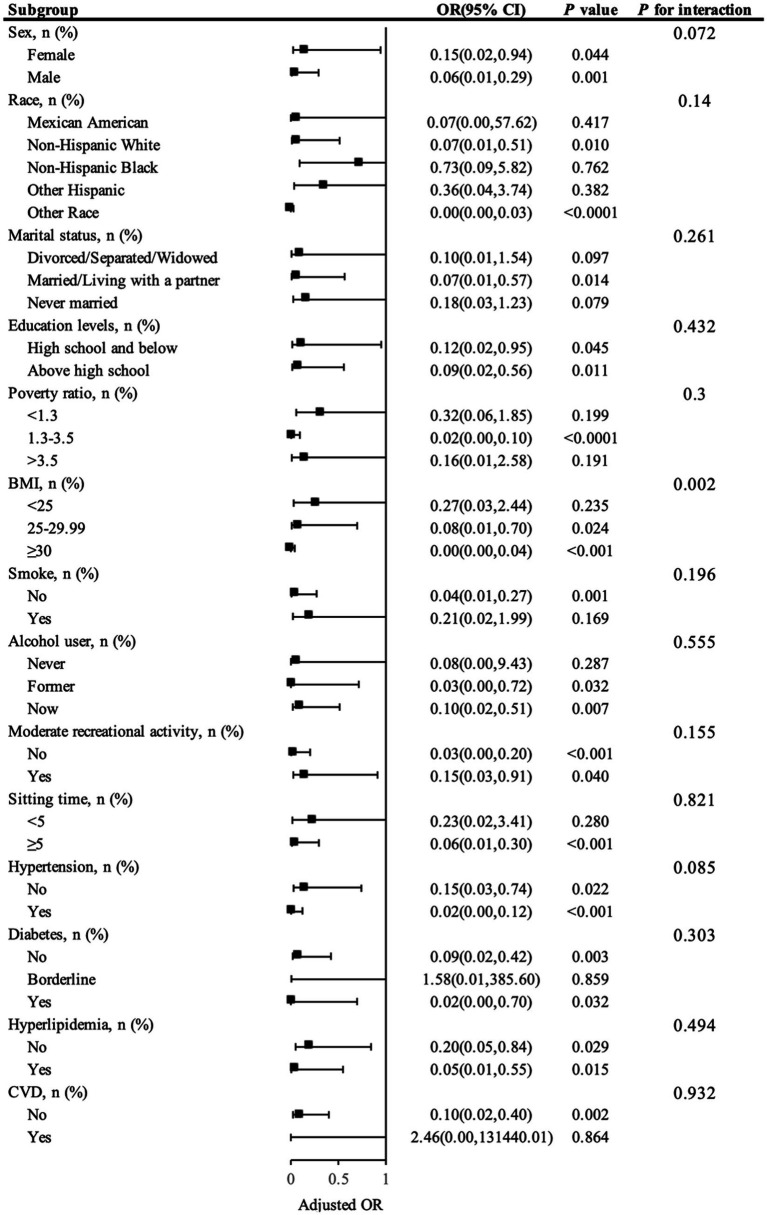
Presents a subgroup analysis of SVR and kidney stone.

Figure 3 illustrates the subgroup analysis, highlighting how SVR correlates with the risk of kidney stones across various demographic categories. In the multivariable model that adjusted for all covariates except the stratifying factors, most subgroups did not show significant variances in the relationship between SVR and kidney stone incidence (interaction *p*-values >0.05). However, it is important to note that the *p*-value for BMI was <0.05, indicating a significant interaction between BMI and SVR. This suggests that BMI plays a significant role in modulating the relationship between SVR and kidney stone risk.

## 4 Discussion

This study is the first systematic exploration of the association between the SVR and kidney stone risk using the NHANES database. The results reveal a significant inverse correlation between SVR and kidney stone risk, indicating that higher SVR is associated with a lower risk of kidney stones. This finding not only supports the positive relationship between muscle mass and renal health but also suggests a detrimental role for visceral fat in the formation of kidney stones, with lower SVR (reflecting higher visceral fat relative to muscle mass) being associated with increased kidney stone risk. These findings provide further evidence for SVR as a potential biomarker to predict kidney stone risk, highlighting the importance of considering overall body composition—particularly the balance between skeletal muscle and visceral fat—when developing clinical prevention strategies for kidney stones.

Firstly, the findings suggest that a higher SVR significantly correlates with a decreased likelihood of developing kidney stones, aligning with prior studies. These studies have linked increased muscle mass with various health benefits, such as enhanced insulin sensitivity, reduced inflammation levels, and increased metabolic rates (10, 12, 17). As a primary organ for glucose and lipid metabolism, increased muscle mass may help stabilize metabolism and reduce the accumulation of substances in urine that could lead to stone formation (17, 18). Additionally, skeletal muscle, being a major site for protein metabolism, offers protective benefits to kidney health. Higher muscle mass has been associated with improved glomerular filtration rates, potentially lowering kidney stone risk (19, 20). Furthermore, skeletal muscle not only serves as an organ for motor output but also plays critical roles in energy metabolism and endocrine regulation (21–23). It secretes various myokines, such as myonectin, which are known to improve insulin sensitivity and reduce inflammation, potentially decreasing the concentrations of calcium and oxalate in urine, thus reducing stone formation (24). Increased muscle mass promotes the uptake of glucose from the bloodstream, helping to maintain normal blood sugar levels, which is important for kidney health (25). Moreover, skeletal muscle is a key regulator of inflammation. Myokines, secreted by muscle tissue, act as anti-inflammatory agents, and their presence reduces the levels of pro-inflammatory cytokines (26). These mechanisms contribute to improved kidney function and may help in reducing the risk of kidney stones by preventing the inflammatory conditions that alter the urine composition, making it more conducive to stone formation. Our results support these findings, demonstrating that an increase in SVR (as a marker of higher muscle mass relative to visceral fat) is significantly associated with reduced kidney stone risk across various quartiles of SVR. This relationship remains robust after adjusting for potential confounders such as age, BMI, and other health factors.

On the other hand, visceral fat, as a metabolically active tissue, secretes various pro-inflammatory and metabolic active substances, like interleukin-6 (IL-6), tumor necrosis factor-alpha (TNF-*α*), and leptin (27, 28), which could potentially elevate the risk of stone formation by enhancing the excretion of calcium and oxalate in the urine and altering its pH and composition (29). Visceral fat’s secretion of pro-inflammatory cytokines and adipokines contributes to systemic inflammation, which can impair kidney function by increasing the levels of oxidative stress. This promotes the formation of urinary crystals and stones (30). Moreover, visceral fat alters the kidney’s ability to concentrate urine, potentially leading to the supersaturation of stone-forming substances like calcium, oxalate, and uric acid (31). These alterations, in combination with an inflammatory environment, create conditions that are highly favorable for kidney stone formation. Therefore, reducing visceral fat may not only lower systemic inflammation but also improve renal function, mitigating the risk of kidney stones. In our analysis, we observed that lower SVR, which corresponds to higher visceral fat relative to skeletal muscle mass, is associated with increased kidney stone risk. This highlights the detrimental role of visceral fat in the development of kidney stones. The significant association between lower SVR and higher kidney stone incidence further underscores the importance of managing visceral fat in kidney stone prevention.

Compared to prior research, a key innovation of this study is the use of SVR as a comprehensive body composition index. Although elevated visceral fat is associated with a heightened risk of kidney stones and higher muscle mass has been shown to potentially reduce this risk, previous studies typically considered the effects of each independently. By integrating these indicators, SVR offers a more comprehensive assessment of body composition, aiding in the more accurate prediction of kidney stone risk. Additionally, improved metabolic states associated with lower excretion of uric acid and calcium salts are directly related to the mechanisms of kidney stone formation (32). Also, a higher muscle mass combined with lower visceral fat may promote better energy utilization and reduced levels of systemic inflammation, thereby lowering the risk of kidney stones.

Additionally, the interaction between BMI and SVR warrants further attention, as our analysis suggests that BMI significantly influences the relationship between SVR and kidney stone risk. Higher BMI, particularly when associated with increased visceral fat, can exacerbate the adverse effects of visceral fat on kidney stone formation (9). Visceral fat, being metabolically active, contributes to systemic inflammation and altered urine composition, both of which can enhance the likelihood of stone formation (33). This highlights that while skeletal muscle mass may confer protective benefits against kidney stones, the positive effects of higher muscle mass may be diminished in individuals with elevated BMI due to the concurrent presence of excessive visceral fat. Thus, BMI should be considered a key factor when interpreting the relationship between SVR and kidney stone risk, emphasizing the importance of managing body fat to mitigate stone formation risk.

Furthermore, the study revealed that the relationship between SVR and kidney stone risk is consistent across different population subgroups, indicating that SVR may be a widely applicable biomarker for assessing kidney stone risk. This is particularly important as it offers a potential screening tool for clinical use to identify high-risk individuals early and potentially mitigate their risk through lifestyle interventions, such as exercise and dietary adjustments.

Nevertheless, this study also presents certain constraints. Given that NHANES is a cross-sectional study, it is not possible to determine causality; specifically, whether an increase in SVR directly leads to a decreased risk of kidney stones. Additionally, while DXA technology, used for assessing body composition, is one of the gold standards, its accuracy may be affected by body shape and posture. Diagnosis of kidney stones relies on self-reporting, which may be subject to recall bias. Moreover, although adjustments were made for various potential confounders, there might still be unaccounted variables that could affect the results. Another potential limitation is the lack of differentiation between different types of kidney stones. Since most kidney stones are calcium oxalate, we recognize that the biochemical and physiological pathways involved in stone formation could differ between stone types.

Future research should validate these findings through a prospective design and explore the relationship between SVR and kidney stone risk across different populations to fully understand its biological basis. Furthermore, exploring the potential of dietary and lifestyle modifications to enhance SVR and consequently prevent the formation of kidney stones remains a crucial avenue for future research. In conclusion, our findings underscore the importance of considering comprehensive body composition indicators in clinical practice, offering new insights and directions for future studies.

## 5 Conclusion

This research, utilizing the NHANES dataset, examined the correlation between SVR and the risk of kidney stones, finding that lower SVR is associated with a higher risk of kidney stones. These findings highlight the possibility of optimizing body composition as a means to prevent kidney stones, providing a biological basis for future targeted prevention measures. Although there are design limitations, these findings provide a foundation for further prospective research and the development of personalized intervention strategies.

## Data Availability

Publicly available datasets were analyzed in this study. This data can be found: The dataset utilized for this analysis can be found within the NHANES database, which is publicly accessible at https://www.cdc.gov/nchs/nhanes/index.htm.
